# Adjunctive amniotic membrane transplantation in the surgical management of fat adherence syndrome: A retrospective clinical series

**DOI:** 10.1097/MD.0000000000049137

**Published:** 2026-05-29

**Authors:** Emine Seker Un, Osman Parca, Tunahan Akyol

**Affiliations:** a Department of Ophthalmology, Göz Akademi Hospital, Denizli, Turkey; b Department of Ophthalmology, Pamukkale University, Denizli, Turkey; c Department of Ophthalmology, Buldan State Hospital, Denizli, Turkey.

**Keywords:** adhesion prevention, amniotic membrane transplantation, fat adherence syndrome, restrictive strabismus, strabismus surgery

## Abstract

This study evaluates the clinical outcomes of adjunctive amniotic membrane transplantation (AMT) in the surgical management of fat adherence syndrome (FAS), a rare but challenging cause of restrictive strabismus following inferior oblique surgery. This retrospective clinical series included patients diagnosed with FAS between 2016 and 2022 who presented with hypotropia, supraduction limitation, and a positive forced duction test after prior inferior oblique surgery. All patients underwent meticulous adhesiolysis, conjunctival recession, and, when indicated, inferior rectus recession, with adjunctive AMT. Surgical success was predefined as postoperative deviation ≤10 prism diopters, improved supraduction without mechanical restriction, and resolution of abnormal head posture. Seven patients (mean age: 17.7 years) met the inclusion criteria. Intraoperatively, dense adhesions involving the inferior rectus muscle, Tenon capsule, and prolapsed fat were identified and successfully released in all cases. Postoperatively, all patients demonstrated improvement in both alignment and motility, and 6 of 7 met the predefined surgical success criterion of ≤10 prism diopters. Diplopia and abnormal head posture resolved in affected patients. No cases of anterior segment ischemia, recurrent restriction, or other complications were observed during 6 to 36 months of follow-up. Adjunctive AMT may help improve postoperative healing and reduce recurrent fibrosis in patients undergoing surgery for FAS. In this retrospective series, favorable functional and cosmetic outcomes were observed following combined surgical management with AMT. Larger prospective controlled studies are required to further clarify its therapeutic contribution.

## 1. Introduction

Fat adherence syndrome (FAS), first described by Parks, is a rare but severe form of restrictive strabismus that can develop following periocular surgery or trauma. It presents as limited ocular motility, hypotropia, marked upgaze restriction, and a positive forced duction test. These clinical findings often progress, resulting in persistent functional limitations.^[1–5]^

FAS most often results from prolapse of orbital fat into the extraconal space due to violation of the posterior Tenon capsule during surgery or from fibrotic adhesions that develop following inflammation-induced loss of elasticity of the extraconal septa. It has been most frequently reported after inferior oblique (IO) muscle surgery, retinal detachment procedures, and lower eyelid blepharoplasty.^[2–4]^

Common treatments include inferior rectus (IR) muscle recession, conjunctival recession (CT), and botulinum toxin injections. Surgical release of fat adhesions often has limited success, and movement restriction may persist.^[1]^

In recent years, amniotic membrane transplantation (AMT) has emerged as a promising adjunct in the management of restrictive strabismus due to its anti-inflammatory and anti-fibrotic properties.^[6]^ This biomaterial supports epithelial cell migration, modulates fibroblast activity, and helps reduce postoperative fibrosis, thereby promoting more favorable healing.^[1]^

Given the challenging nature of FAS and the limited success of conventional approaches, this study evaluates the clinical outcomes of adjunctive AMT in the surgical management of FAS.

## 2. Methods

This study retrospectively reviewed the clinical records of all patients diagnosed with FAS between 2016 and 2022.

Patients who developed restrictive strabismus after IO muscle surgery and were clinically diagnosed with FAS based on hypotropia, supraduction limitation, and a positive forced duction test were included. Clinical characteristics – including age, sex, laterality, prior ocular surgeries, pre- and postoperative vertical deviation (prism diopters [PD]), presence of diplopia, cosmetic concerns, and abnormal head posture – were retrospectively reviewed.

In all cases, restrictive adhesions were surgically released through meticulous dissection of adherent fat and fibrotic Tenon tissue. AMT was applied in conjunction with CT, and IR recession was added when indicated based on preoperative deviation and intraoperative findings. The amniotic membrane was trimmed according to the size of the dissected area and placed with the stromal side facing the scleral surface. The graft was positioned to cover the exposed IR muscle, surrounding Tenon tissue, and the area of released adhesions. Fixation was achieved using interrupted 8-0 Vicryl sutures to the adjacent conjunctival edges. Care was taken to avoid graft folding or excessive tension during placement. Cryopreserved amniotic membrane was applied over the dissected area as a biological barrier, with attention to graft size, placement, and fixation to adequately cover the exposed tissues. Forced duction testing was performed preoperatively and postoperatively to assess mechanical restriction. All surgeries were performed by a single experienced strabismus surgeon using a standardized operative technique.

Postoperatively, all patients received hourly topical antibiotic–steroid therapy for the first 3 days, followed by a gradual taper over 6 weeks. In addition, systemic corticosteroids (0.5 mg/kg/day) were administered for 4 weeks to minimize inflammation and reduce the risk of recurrent adhesions.

Surgical success was defined as the presence of no clinically significant deviation (≤10 PD), improved supraduction without mechanical restriction, and resolution of abnormal head posture. Supraduction limitation was graded on a clinical scale ranging from −1 (mild limitation) to −4 (severe limitation). Forced duction testing was recorded qualitatively as indicating the presence or absence of mechanical restriction, and a formal graded scale was not available in this retrospective series.

Due to the small sample size and the descriptive nature of this retrospective case series, no formal statistical analysis was performed.

The study was approved by the local ethics committee and conducted in accordance with the principles of the Declaration of Helsinki.

## 3. Results

Seven patients who developed FAS following IO surgery were included. The mean age at presentation was 17.7 years (range: 4–44 years). Baseline demographic and clinical features are summarized in Table 1. All patients exhibited hypotropia, marked supraduction limitation, and a positive preoperative forced duction test.

**Table 1:** Demographic and clinical data.

Case	Age/sex	Previous surgical procedure	Presenting findings	Hypotropia at presentation	Elevation limitation	Diplopia	Abnormal head position
1	21 yr/M	Bilateral 5.5 mm MR recession + right IO myectomy + right 6 mm LR resection	Left 12 PD hypertropia + 26 PD exotropia, right 12 PD hypotropia	12 PD	−4	No	No
2	4 yr/F	Bilateral 6 mm LR recession + bilateral IO myectomy	Left 8 PD hypertropia + 20 PD esotropia, left 8 PD hypotropia	8 PD	−2	No	No
3	44 yr/F	Unknown (likely bimedial recession + IO myectomy)	Right 30 PD hypertropia + 18 PD esotropia, left 30 PD hypotropia	30 PD	−4	No	No
4	6 yr/M	Left 5 mm MR recession + right 6.5 mm LR resection + right IO myectomy	Left 14 PD hypertropia + 26 PD exotropia, right 14 PD hypotropia	14 PD	−2	No	No
5	2 yr/M	Bilateral 7 mm LR recession + right IO myectomy	Right 12 PD hypertropia, left 10 PD hypotropia	12 PD	−2	Yes	Yes
6	<1 yr/F	Bilateral 5.5 mm MR recession + bilateral IO myectomy	Right 10 PD hypertropia + 45 PD exotropia, left 10 PD hypotropia	10 PD	−3	No	No
7	7 yr/F	Left 7 mm LR recession + left 6 mm MR resection + left IO myectomy	Left 12 PD hypertropia + 30 PD exotropia, right 12 PD hypotropia	12 PD	−2	No	No

IO = inferior oblique, LR = lateral rectus, MR = medial rectus, PD = prism diopters.

Most patients were referred for cosmetic concerns, whereas diplopia and abnormal head posture were present only in those who retained binocular vision. All cases showed unilateral involvement and had documented or clinically suggestive evidence of prior IO muscle surgery.

Intraoperatively, dense adhesions involving the IR muscle, its fascial sheath, and adjacent prolapsed fat were consistently encountered and released. All patients underwent IR recession, and additional horizontal muscle procedures were performed when indicated. CT and AMT were applied in every case. Detailed surgical procedures and postoperative alignment findings are presented in Table 2.

**Table 2:** Surgical procedures and postoperative outcomes.

Case	Surgical procedure	Postop deviation	Postop elevation limitation	Postop diplopia	Abnormal head position	Follow-up duration
1	Right IR 3 mm recession + CT + AMT + left LR 7 mm resection	2 PD left hypertropia + 12 PD exotropia	−2	No	No	6 mo
2	Right IR 2.5 mm recession + left IO myectomy + left MR 4 mm recession + CT + AMT	None, 10 PD esotropia	−1	No	No	6 mo
3	Left IR 3 mm recession + left IO disinsertion + right LR 5 mm recession + CT + AMT	Right 10 PD hypertropia + 8 PD esotropia	−2	No	No	26 mo
4	Right IR 3 mm recession + CT + AMT + left MR reinsertion	Left 4 PD hypertropia + 6 PD exotropia	−1	No	No	15 mo
5	Right IR 3 mm recession + right IO disinsertion + left MR 7 mm resection + CT + AMT	Left 6 PD hypertropia + 14 PD exotropia	−1	No	No	18 mo
6	Left IR 3 mm recession + AMT + CT + bilateral MR reinsertion	Right 4 PD hypertropia + 12 PD exotropia	−2	No	No	9 mo
7	Left IR 2.5 mm recession + AMT + CT	Right 4 PD hypertropia	−1	No	No	36 mo

AMT = amniotic membrane transplantation, CT = conjunctival recession, IO = inferior oblique, IR = inferior rectus, LR = lateral rectus, MR = medial rectus, PD = prism diopters.

Postoperatively, all patients demonstrated meaningful improvement in both alignment and supraduction. The mean preoperative vertical deviation was 14.0 ± 7.2 PD (range: 8–30 PD), which decreased to 4.3 ± 3.0 PD (range: 0–10 PD) postoperatively, reflecting a substantial reduction compared with preoperative measurements. Six of 7 patients achieved alignment within the predefined surgical success threshold, whereas 1 patient exhibited a mild residual deviation that remained stable throughout follow-up.

No patient developed anterior segment ischemia, recurrent restriction, new-onset diplopia, or abnormal head posture. Follow-up durations ranged from 6 to 36 months, and no postoperative complications were observed during this period.

## 4. Discussion

FAS is a rare but clinically severe cause of restrictive strabismus that may develop after IO surgery. In our series, AMT served as a valuable adjunct to adhesiolysis and IR recession, thereby reducing inflammation and fibrosis. All patients demonstrated postoperative improvement in alignment and motility, and 6 of 7 met the predefined surgical success criterion of ≤10 PD. Patients with diplopia and abnormal head posture also showed symptomatic improvement.

The incidence of FAS has been reported with varying rates. Parks identified a 2% rate after IO surgery, whereas later series reported up to 13% after myectomy.^[7,8]^ Recent literature suggests a decreasing incidence with enhanced anatomical understanding and improved surgical technique. Nevertheless, FAS is not exclusively related to surgical error; it has been documented even after appropriately performed procedures.^[9–11]^ Careful evaluation of ocular motility, intraoperative anatomy, and forced duction testing remains crucial. Because all patients in our study were referrals to a tertiary center, incidence estimation was not possible.

The existing literature on FAS is limited and primarily consists of small case series. Merino et al reported partial improvement after adhesiolysis, but complete motility recovery was rare. In contrast, our findings demonstrated a more pronounced improvement, with deviation decreasing from 14 to 4.29 PD and supraduction improving in all cases, suggesting that AMT may have contributed to preventing recurrent fibrosis.^[12]^ Residual horizontal deviations observed postoperatively in some patients largely reflected preexisting complex strabismus patterns rather than newly induced postoperative misalignment. Several patients had substantial horizontal deviations preoperatively, including exotropia or esotropia associated with long-standing ocular motility imbalance following prior strabismus surgery. In these cases, individualized horizontal muscle procedures were performed when clinically indicated as part of the overall surgical strategy. Although improvement in vertical alignment and supraduction was achieved in all patients, small residual horizontal deviations persisted in certain cases, likely due to the chronic and multifactorial nature of the underlying strabismus. Amniotic membrane may provide biological advantages by acting as a physical barrier and modulating inflammation and fibroblast activity, thereby reducing postoperative fibrosis and adhesion formation.

Previous studies, including those by Yamada and Shinoda, support the use of AMT in preventing readhesion in restrictive strabismus following retinal surgery.^[13]^ Beyond restrictive strabismus, AMT’s anti-inflammatory, anti-fibrotic, and epithelial healing effects have been demonstrated across various ocular surface pathologies, with favorable outcomes in multiple series.^[14–16]^ Comparative data also suggest that AMT has a safer profile than mitomycin C, which carries risks of inflammation and tissue toxicity.^[17,18]^ Although botulinum toxin injections have been proposed for acute-phase FAS, none of our patients presented during this period.^[19,20]^ In clinical practice, adjunctive AMT may be particularly considered in patients with severe adhesions, recurrent restriction, or high risk of postoperative fibrosis following prior IO surgery.

Overall, the addition of localized AMT to conventional surgery appears to reduce recurrent adhesions and enhance healing, thereby improving alignment, motility, and cosmetic outcomes. Prevention remains the most effective approach; preserving posterior Tenon integrity, avoiding orbital fat exposure, and ensuring meticulous hemostasis are essential intraoperative principles. For patients who develop FAS, realistic expectations should be discussed preoperatively, as full functional recovery cannot always be achieved. Although no recurrence was observed during follow-up, the minimum follow-up duration of 6 months may not have been sufficient to detect late adhesion recurrence in all cases.

This series offers a unique retrospective clinical setting, with all surgeries performed by a single experienced surgeon and adjunctive AMT applied uniformly, thereby strengthening the interpretability of postoperative outcomes. As all cases of FAS were referred to our hospital from other centers, the interval between the original IO surgery and the corrective procedure could not be consistently determined, which may have influenced adhesion severity and postoperative outcomes. However, several limitations should be acknowledged. The retrospective design and small sample size – an inherent challenge when studying a rare condition such as FAS – limit the generalizability of the results. The lack of a control group treated without AMT prevents definitive conclusions about its comparative efficacy, and variability in prior surgical techniques at referring institutions may have influenced adhesion severity and postoperative outcomes. Given the combined surgical approach, including adhesiolysis, muscle recession, and systemic corticosteroid therapy, the specific contribution of AMT cannot be determined independently. These limitations underscore the need for larger, controlled, prospective studies to clarify the role of AMT in FAS management.

## 5. Conclusion

FAS is an uncommon but clinically challenging complication of IO surgery, often resulting in persistent restrictive strabismus despite appropriate management. Our findings suggest that adding AMT to standard adhesiolysis and IR recession may help reduce inflammation and limit recurrent fibrosis, leading to meaningful improvements in alignment and motility in most patients. However, because complete resolution is not always achievable, realistic preoperative expectations must be established. Prevention remains critical, and meticulous preservation of posterior Tenon integrity and avoidance of orbital fat exposure are essential. Although adjunctive AMT appears to provide therapeutic benefit, larger prospective studies are required to confirm its role and define standardized treatment algorithms.

## Acknowledgments

The authors would like to thank the medical and administrative staff of Pamukkale University for their support throughout the study. This study was also presented as an oral presentation at the 45th Turkish Ophthalmological Association (TOD) Winter Symposium (2024) under the title “Şaşılık cerrahisi sonrası gelişen yağ adezyon sendromlu hastalarda tedavi ve postoperatif sonuçlarımız” (Oral Presentation No. SS-50).

## Author contributions

**Conceptualization:** Osman Parca.

**Project administration:** Emine Seker Un.

**Data curation:** Tunahan Akyol.

**Methodology:** Tunahan Akyol.

**Supervision:** Emine Seker Un.

**Investigation:** Osman Parca.

**Software:** Osman Parca.

**Validation:** Osman Parca.

**Writing – original draft:** Tunahan Akyol.

**Writing – review & editing:** Emine Seker Un.

## Correction

The originally published acknowledgement section has now been revised as follows; From “The authors would like to thank the medical and administrative staff of Buldan State Hospital and Pamukkale University for their support throughout the study” To “The authors would like to thank the medical and administrative staff of Pamukkale University for their support throughout the study. This study was also presented as an oral presentation at the 45th Turkish Ophthalmological Association (TOD) Winter Symposium (2024) under the title “Şaşılık cerrahisi sonrası gelişen yağ adezyon sendromlu hastalarda tedavi ve postoperatif sonuçlarımız” (Oral Presentation No. SS-50).”
